# Emotional Distress Following Amyloid PET Disclosure: A Preliminary Analysis Across Cognitive Spectrum

**DOI:** 10.1002/alz.086875

**Published:** 2025-01-03

**Authors:** Jeong Eun Kim, Dianxu Ren, Annie Cohen, Oscar L. Lopez, Jennifer H Lingler

**Affiliations:** ^1^ School of Nursing, University of Pittsburgh, Pittsburgh, PA USA; ^2^ University of Pittsburgh, Pittsburgh, PA USA; ^3^ University of Pittsburgh Alzheimer’s Disease Research Center (ADRC), Pittsburgh, PA USA; ^4^ University of Pittsburgh School of Medicine, Pittsburgh, PA USA; ^5^ Department of Psychiatry, University of Pittsburgh, Pittsburgh, PA USA; ^6^ University of Pittsburgh Alzheimer’s Disease Research Center, Pittsburgh, PA USA

## Abstract

**Background:**

Gaining insight into the emotional consequences of disclosing amyloid Positron Emission Tomography (PET) results is essential for offering effective support to patients of varying cognitive status. This analysis aimed to examine variations in emotional distress levels following the disclosure of amyloid PET results among participants that are cognitively normal, with Mild Cognitive Impairment (MCI), or dementia.

**Method:**

This investigation was a preliminary analysis of 55 participants, using 1‐month follow‐up call data of an ongoing brain imaging data repository study, obtaining baseline PET imaging on a subset of Alzheimer’s Disease Research Center (ADRC) participants. Multiple linear regression modelling was used to examine the relationship between emotional distress scores, measured by Impact of Genetic Testing questionnaire modified for amyloid PET disclosure, and diagnostic categories, while controlling for participants’ sex, age, level of education, and amyloid status. Additionally, pairwise comparisons were conducted to examine specific differences between diagnostic categories. Bonferroni correction was applied to adjust for multiple comparisons.

**Result:**

Participants were predominantly White (94.5%), Non‐Hispanic (94.5%), highly educated (average of 16.6 years) individuals, with a mean age of 70.8. Of the 55 participants, 13 were cognitively normal, 17 with MCI, and 25 diagnosed with dementia. Approximately 54% were amyloid negative. During the 1‐month follow‐up call, participants exhibited a mean depression score (measured by Center for Epidemiological Studies Depression Scale) of 6.93 (SD = 7.57) and a mean anxiety score (measured by Spielberger State‐Trait Anxiety Inventory) of 28.55 (SD = 10.56). Participants’ diagnostic categories were marginally significantly associated with their emotional distress scores post disclosure (b = 4.945, SE = 2.577, p = 0.062). However, individual diagnostic categories revealed a positive direction in mean emotional distress scores: normal cognition (M = 7.320, SE = 4.306), MCI (M = 16.955, SE = 3.382), and dementia (M = 18.572, SE = 2.791).

**Conclusion:**

These preliminary findings suggest that the disclosure of amyloid PET results does not result in discernible differences in the level of emotional distress among cognitively symptomatic and asymptomatic individuals. This contributes to our understanding of the emotional impact of amyloid PET disclosures and may have implications for counseling and support strategies in the context of Alzheimer’s disease research and clinical settings. Further investigation with a larger sample size is recommended to validate these findings.

